# Antiangiogenic Therapy for Glioma

**DOI:** 10.1155/2012/483040

**Published:** 2012-07-08

**Authors:** Valentina Cea, Carlo Sala, Chiara Verpelli

**Affiliations:** ^1^Department of Pharmacology, CNR Institute of Neuroscience, University of Milan, Via Vanvitelli 32, 20129 Milan, Italy; ^2^Neuromuscular Diseases and Neuroimmunology, Neurological Institute Foundation Carlo Besta, Via Celoria 11, 20133 Milan, Italy

## Abstract

Currently, antiangiogenic agents are routinely used for the treatment of patients with glioma. However, despite advances in pharmacological and surgical therapy, glioma remains an incurable disease. Indeed, the formation of an abnormal tumor vasculature and the invasion of glioma cells along neuronal tracts are proposed to comprise the major factors that are attributed to the therapeutic resistance of these tumors. The development of curative therapeutic modalities for the treatment of glioma requires further investigation of the molecular mechanisms regulating angiogenesis and invasion. In this review, we discuss the molecular characteristics of angiogenesis and invasion in human malignant glioma, we present several available drugs that are used or can potentially be utilized for the inhibition of angiogenesis in glioma, and we focus our attention on the key mediators of the molecular mechanisms underlying the resistance of glioma to antiangiogenic therapy.

## 1. Introduction

Angiogenesis and tumor cell invasion play a critical role in glioma development and growth, even during the earliest phases [1]. Indeed, the formation of abnormal tumor vasculature and glioma cell invasion along white matter tracts are proposed to be the major causes of the therapeutic resistance of these tumors; thus, glioma remains a fatal disease despite advances in surgical and medical therapy. 

Glioma tumors are an example of highly vascularized tumors, which induce angiogenesis by upregulating vascular endothelial growth factor (VEGF) and its downstream pathways. Indeed, several molecular abnormalities have been described in glioma that promote angiogenesis, such as mutations and/or upregulation of PI3K/Akt and the VEGF receptor (VEGFR) in the glioma endothelium [2]. Interestingly, each of these signaling pathways involves alterations that can be therapeutically targeted [3]. Evaluation of drugs that target these pathways requires novel preclinical and clinical experimental trial design to define the optimal drug dose and delivery times to avoid toxicity during the first months of treatment [4, 5]. Furthermore, whether these agents can be used in combination with classical cytotoxic chemotherapy, what molecular markers can predict response, and whether they can be potentiated by such combinatorial treatments are important issues that remain to be explored. 

In this paper, we first discuss the molecular characteristics of angiogenesis and invasion in human malignant glioma. Secondly, we discuss the commercially available drugs that are currently used or might be potentially utilized for the inhibition of angiogenesis in glioma. Thirdly, we focus our attention on the key mediators of the molecular mechanisms underlying the resistance that glioma exhibits to antiangiogenic therapy. Finally, we highlight the necessity for further investigation of the clinical utility of antiangiogenic therapies and the development of novel strategies for the treatment of glioma.

## 2. Angiogenesis in Glioma

Angiogenesis, the formation of new blood vessels, is a critical step during tumorigenesis and represents a pathological hallmark of cancer. 

When a solid tumor, such as a brain tumor, grows larger than a critical size (1-2 mm in diameter), it must recruit new blood vessels to supply the required oxygen and nutrition levels necessary for its survival and proliferation. This process comprises the formation of new blood vessels from preexisting ones and is a crucial step in the progression of cancer from a small and localized neoplasm to a highly aggressive tumor. The major *in vitro *and *in vivo *models to study tumor angiogenesis are summarized and critically discussed in Table 1.

### 2.1. Mechanisms of Neoangiogenesis in Glioma

Angiogenesis requires three distinct steps: (1) blood vessel breakdown, (2) degradation of the vessel basement membrane and the surrounding extracellular matrix (ECM), and (3) migration of endothelial cells and the formation of new blood vessels.

Angiogenesis plays a crucial role in glioma development and growth [1]. Gliomas are highly vascularized tumors and neovascularization in and around the tumor are well characterized. Holash et al. reported also vascular cooption before the real agiogenesis in an experimental model of glioma [28]. The level of angiogenesis is correlated with the aggressiveness of gliomas and is often associated with prognosis. Glioblastomas (GBM) are the most lethal cancer and the most vascularized brain cancer, with the highest degree of vascular proliferation and endothelial cell hyperplasia [29]. Patients with high tumor microvascular densities exhibit shorter postoperative survival rates than patients with low microvascular densities [30, 31]. Perivascular migration, proliferation, and angiogenesis are closely associated and progress concurrently in gliomas.

### 2.2. Angiogenic Factors

Angiogenesis results from a balance between proangiogenic factors and antiangiogenic factors. During tumor progression, there is a shift towards angiogenic factors that stimulate uncontrolled and disorganized vascular growth. These molecular factors can be secreted by cancer, endothelial, stromal, and blood cells and by the extracellular matrix [32, 33].

Proangiogenic factors involve a variety of angiogenic factors including vascular endothelial growth factor (VEGF), acidic fibroblast growth factor, basic fibroblast growth factor, placental growth factor, angiopoietin-2, and interleukins, whereas antiangiogenic factors include angiostatin, endostatin, thrombospondin 1, and endothelial monocyte-activating polypeptide 2 [33, 34]. In addition, enzymes including serine proteinases and metalloproteinases degrade the extracellular matrix, which plays an important role in both the induction and the suppression of angiogenesis [35]. For example, Raithatha et al. [36] reported that MMP-9 might regulate angiogenic remodeling. Autocrine or paracrine factors of the glioma microenvironment and PDGF also contribute to angiogenesis in gliomas [37]. The endothelial cells that are stimulated by angiogenic factors then migrate and proliferate, resulting in neovascular formation [21]. This paracrine loop constitutes an extended foothold that allows tumor cells to migrate.

### 2.3. The VEGF/VEGFR Pathway

Accumulating evidence indicates that the VEGF and VEGFR signaling pathways play a major role in tumor angiogenesis in malignant glioma, similar to most other solid tumors. 

VEGF-A is upregulated in glioblastoma and is produced by multiple cell types, including the tumor, stromal, and inflammatory cells [38]. The expression levels of VEGF are regulated by the following mechanisms: (1) low oxygen concentrations in growing gliomas induces upregulation of HIF that increases VEGF mRNA levels; (2) EGFR signaling stimulates *VEGF* gene expression via an HIF-independent mechanism; (3) FoxM1B transcription factor is upregulated in glioblastoma multiforme and stimulates VEGF expression independently of HIF [39]; (4) upregulation of the HuR protein that suppresses the posttranscriptional degradation of *VEGF-A* mRNA under hypoxia leads to a further increase in VEGF levels [40]; (5) brain-derived neurotrophin factor can enhance the expression of VEGF, increasing the levels of hypoxia-inducible factor-1 expression [41]; (6) integrin-linked kinase 1(ILK1) is an important regulator of tumor angiogenesis because it increases VEGF expression by stimulating HIF-1*α* via AKT phosphorylation on Ser^473^ [42].

The two tyrosine kinase receptors, VEGFR-1 and VEGFR-2, are both highly expressed in gliomas [43]. They are activated by VEGF-A but are differentially linked to angiogenesis and glioma growth *in vivo*. VEGFR-2 is mainly expressed in vascular endothelial cells, where it directly transduces most of the mitotic signals that result in angiogenesis. In contrast, VEGFR-1 is expressed not only in vascular endothelial cells but also in monocyte/macrophage lineage cells. These macrophages can act as proangiogenic and protumorigenic cells, similar to the tumor-associated macrophages [44, 45].

VEGFR induces the activation of different signaling pathways including activation of Ras/Raf/mitogen-activated protein kinase [46, 47] and phospholipase C-*γ*/protein kinase C [47], which regulate endothelial cell proliferation and migration [48]. VEGF also enhances vascular permeability through the MAPK signaling cascade by rearranging cadherin/catenin complexes and perturbing the adherens junctions between endothelial cells [49, 50]. Another important signaling cascade activated not only by VEGFR but also by other proangiogenic stimuli, including platelet-derived growth factor, neurotrophins, insulin-like growth factor, epidermal growth factor, and integrins, is the phosphatidylinositol-3 kinase/AKT pathway [51, 52], that is, fundamentally altered during brain tumor angiogenesis [53]. 

Notably, brain tumor blood vessels are tortuous, disorganized and highly permeable, resulting in irregular and inefficient blood flow [54–57] and vasogenic brain edema. This irregularity and inefficiency are strongly associated with the action of VEGF. 

One of the insidious features of gliomas is their ability to metastasize and to establish numerous microtumors at a distance from the primary tumor. In all types of gliomas, the potential of single cells to invade normal brain tissue is closely related to angiogenesis. 

The formation of abnormal tumor vasculature and glioma cell invasion along white matter tracts are believed to be the major factors responsible for the resistance of gliomas to treatment. Therefore, further investigation of the mechanisms underlying angiogenesis and invasion in glioblastoma is essential for the development of a curative therapy.

## 3. Antiangiogenic Therapy for Glioma

Tumors require nutrients and oxygen in order to grow, and new blood vessels provide these requirements. GBM cells are characterized by their invasive abilities and striking angiogenic potential. The blood vessels formed by tumor cells are structurally and functionally abnormal: the blood vessels are leaky and dilated, the endothelial cells exhibit aberrant morphology, the pericytes are loosely attached or absent and the basement membrane is incomplete [14]. These abnormalities lead to an abnormal tumor microenvironment that is characterized by interstitial hypertension, hypoxia and acidosis. The abnormal vasculature represents a barrier to the delivery and efficacy of anticancer therapeutic agents. These observations suggest that if the structure and function of tumor vessels could be “corrected,” then the tumor microenvironment might be normalized, ultimately improving the efficacy of cancer treatments.

As a key mediator of angiogenesis, VEGF and its receptors are targets for anticancer therapies [58], in addition to conventional therapies. Targeting the cells that support tumor growth, rather than the actual tumor cells, represents a relatively new approach to cancer therapy. This approach is particularly promising because these support cells are genetically stable and therefore less likely to develop mutations that will allow them to develop drug resistance in a rapid manner.

A significant challenge for antiangiogenic therapy is to design combination protocols that can counteract the diverse angiogenic stimuli produced by the tumor and its microenvironment.

VEGF signaling inhibitors have been shown to significantly suppress or delay tumor growth in several animal models [59] and in clinical trials. The humanized monoclonal anti-VEGF antibody bevacizumab is the first VEGF-targeting drug approved for use in patients with metastatic colorectal cancer [60], metastatic breast cancer, lung cancer, renal cell carcinoma, and glioblastoma multiforme [61].

VEGF expression is regulated by intrinsic and extrinsic factors. Hypoxia and hypoglycemia are major stimulators of VEGF expression [62]. Factors that can potentiate VEGF production and stimulate angiogenesis include tumor necrosis factor and transforming growth factor.

Several approaches have been used to eliminate the hypoxic cells within tumors [63].

### 3.1. Antiangiogenic Strategies

Angiogenesis inhibitors have been divided into two classes: direct and indirect [64]. Direct angiogenesis inhibitors, such as endostatin, target the microvascular ECs, preventing their response to various proangiogenic stimuli and thereby enhancing the effects of chemotherapy.

In contrast, indirect angiogenesis inhibitors interfere with the proangiogenic communication between the tumor cells and the endothelial cell compartments. Antiangiogenic therapies act predominantly by blocking the binding of VEGF to its receptor and comprise neutralizing antibodies against the ligand or the receptor, soluble receptors, or small molecule inhibitors directed against the tyrosine kinase activity of the VEGF receptors.

Due to the potential of tumor “escape” when specific, indirect antiangiogenic agents (e.g., anti-VEGF) are delivered individually, appropriate combination protocols employing these agents are required for maximal benefit [65]. Abdollahi and coworkers show that the treatment of tumor xenografts with a combination of endostatin and with VEGF blockers results in an enhanced therapeutic effect, which may be attributed to the endostatin-mediated downregulation of many regulators of proangiogenic pathways and suppression of alternative angiogenic mechanisms that might be upregulated by VEGF blockade [66].

Here, we focus on several molecules that interfere with the VEGF/VEGFR signaling pathway, which have been evaluated in clinical trials for solid tumors. In Table 2, we summarize the available treatments and the relative clinical phases and results.

### 3.2. Indirect Antiangiogenic Drugs

As mentioned previously, Bevacizumab (Avastin) is a humanized neutralizing monoclonal antibody that blocks the binding of human VEGF to its receptors. A significant tumor response was observed in response to Bevacizumab treatment: the 6-month progression-free survival was 32% in GBM patients [84]. However, glioblastoma appears to adapt rapidly to anti-VEGF therapy, resulting in rapid tumor progression without improvement in overall survival [85, 86].

In recent study demonstrated that anti-VEGF therapies can significantly reduce the vascular supply, as demonstrated by a decrease in intratumoral blood flow and a strong reduction of large- and medium-size blood vessels, however these events were also shown to be accompanied by a strong increase in infiltrating tumor cells in adjacent brain parenchyma [87]. Finally, a preclinical study [88] and a clinical trial [89] suggest that high doses of bevacizumab could directly enhance the invasiveness of human glioblastoma cell lines and that dosages lower than those currently used might improve patient outcome. 

In the endothelial cells of normal animals, VEGF-A treatment results in the upregulation of both integrins *α*1*β*1 and *α*2*β*1. The functional blocking of these integrins impairs angiogenesis *in vitro* and reduces VEGF-A-induced angiogenesis and tumor growth *in vivo* [90, 91]. *α*v*β*3 integrins are highly expressed by proliferating and activated vascular endothelial cells. Therefore, they are a major contributor to the formation of vasculature by supporting the migration and survival of endothelial cells [92]. The blockade of *α*v*β*3 integrins inhibits tumor angiogenesis as well as blood vessel formation in *in vivo* models [93, 94]. Consequently, *α*v*β*3 might represent a potential target in antiangiogenic therapy. Antagonizing integrins has generally included the targeting of the receptor binding sites or other nearby sites, although new alternative approaches target downstream signaling proteins.

Cilengitide is a cyclic RGD-peptide inhibitor of *α*v*β*3 and *α*v*β*5 integrins. Blocking *α*v*β*3 integrin inhibits blood vessel formation *in vivo* [95]. In a phase II trial, cilengitide was associated with a median survival of 10 months in recurrent glioma patients [96]. Cilengitide is currently in clinical phase III studies for the treatment of glioblastomas and is in phase II studies for the treatment of several other tumor types, including breast cancer, squamous cell cancer, nonsmall cell lung cancer, and melanoma [97, 98].

Other drugs targeting integrins include the following agents.

Abergrin is a humanized antibody against *α*v*β*3 integrins. It blocks integrin binding to vitronectin and fibrinogen, preventing cell adhesion, migration, proliferation, and integrin-mediated cell signaling [99].

Volociximab is a chimeric human-mouse monoclonal antibody that binds to *α*5*β*1 integrins. It induces cell death and prevents capillary tube formation *in vitro*. *In vivo, *volociximab exhibits antitumor and antiangiogenic effects [100].

Increased matrix metalloproteinase (MMP) levels are associated with glioma invasion and angiogenesis. Marimastat reduces MMP levels in patients with gliomas [73]. Phase II clinical trials evaluating the administration of marimastat in combination with temozolomide demonstrated promising results (the progression-free survival after six months was 39%), although further investigation is needed for the associated therapy-induced joint pain [101].

Sorafenib (Nexavar) is a multi-kinase inhibitor of VEGFR2-3, PDGFR, Raf kinase, and c-Kit. It is currently approved for the treatment of advanced HCC and renal cell carcinoma. Phase II trials evaluating the efficacy of sorafenib in patients with malignant glioma are currently ongoing [102]. Hypertension is a specific side effect of sorafenib and of most antiangiogenic agents due to the decreased production of nitric oxide and prostacyclins in vascular endothelial cells [103].

Cediranib (Recentin) is a potent inhibitor of both VEGFR-1 and VEGFR-2. It also exhibits activity against c-kit, PDGFR-beta and FLT4. It is well tolerated, and an inverse correlation was found between cediranib dose- and time-dependent treatment and soluble VEGFR-2 [104].

Sunitinib (Sutent) is a multi-kinase inhibitor of VEGFR 1-3, RET and PDGFR, approved for treatment of RCC, imatinib-resistant gastrointestinal stromal tumors (GIST) and pancreatic neuroendocrine tumors (pNET) [105–107]. A recent preclinical study [108] shows that after starting sunitinib treatment, there is a period when tumor oxygenation is higher in treated compared to untreated mice. The improved oxygenation suggests that the residual blood vessels had improved function in terms of delivering oxygen and nutrients. A synergistic delay in tumor growth was observed when radiation was applied during the enhanced tumor oxygenation after 4 days of sunitinib administration.

Imatinib is a kinase inhibitor of PDGFR, c-kit, and bcr-abl. Administration of imatinib at low concentrations can act as a cytostatic agent, whereas at high concentrations, it predominantly behaves as a cytotoxic agent [109]. Imatinib monotherapy has failed due to the limited penetration of the drug across the BBB, and for that reason, the inhibition of PDGFR alone is insufficient to prevent the growth of malignant gliomas [110]. 

Antiangiogenic therapies are integrated into the treatment strategies for many different tumor types. However, not all patients respond to therapy; only a few benefit with progression-free survival. In most tumors, antiangiogenic treatment is combined with chemotherapy. Furthermore, a major problem of this therapy is the development of resistance. Extensive evidence indicates that antiangiogenic therapy might actually enhance tumor progression by promoting an invasive phenotype that allows for tumor cells to escape angiogenic inhibition.

The identification of predictive biological markers of objective response will be critical for the assessment of the response rates correlated with overall survival and of the development of resistance to antiangiogenic drugs. These markers will provide important indices to aid in the improvement of therapeutic efficacy or in the development of alternative antiangiogenic therapies in the event of treatment failure. 

## 4. Molecular Mechanisms of Resistance to Antiangiogenic Therapy in Glioma

VEGF is ubiquitously expressed in almost all tumors. Tumor cells have been demonstrated to secrete VEGF, which leads to increased angiogenesis [111, 112].

Although antiangiogenic treatment yields survival benefits for patients with many different types of aggressive tumors, VEGF pathway inhibitors are nonetheless failing to produce enduring clinical responses in most patients [113, 114]. Here, we discuss the different ways that tumors can circumvent antiangiogenic therapy.

### 4.1. Alternative Pathway Activation by Tumoral Cells

One way that tumor cells bypass antiangiogenic therapy is via the activation or upregulation of alternative proangiogenic pathways. In preclinical models and in clinical trials, overexpression of fibroblast growth factor 1 and 2, ephrin, and angiopoietin was found in tumors that were treated with inhibitors of VEGF signaling [115]. 

Pericytes play an important role in the pathology of aberrant tumor vasculature. The vessels within tumors that survive antiangiogenic therapy are tightly covered with pericytes, which are recruited by vascular endothelial cells to provide VEGF, the most important survival signal for endothelial cells [116]. Important features of hypoxic remodeling include the loss of small vessels and extensive proliferation of vascular mural cells (MCS) in the surviving vasculature. PDGF-B appears to play a significant role in promoting the integrity of vascular networks during conditions of environmental stress. Recruited MCs are key contributors to the maintenance of tumor neovasculature. PDGF-B signaling via the PDGF receptor-*β* (PDGFR-*β*) plays a critical role in MC recruitment [117]. Similar to VEGF, PDGF-B expression in ECs is critically regulated by oxygen tension, and PDGF-B overexpression is associated with abnormal proliferation of MCs [118]. Members of the ephrin family have been shown to play important roles in regulating the assembly of vascular cells.

However, increased PDGF-B expression has only been found in recurrent xenografts. PDGFR-*β* was also found in the large vessels of the recurrent tumors. Prolonged antiangiogenic therapy significantly alters the expression of angiogenic factors implicated in vascular MC recruitment, causing extensive morphological changes in vessels, including significant increases in diameter and active proliferation of vascular mural cells [119].

Cancer cells can also adapt to the disruption of vessels by extravagating into normal tissues [120].

An alternative mechanism of escaping from VEGF blockade might be attributed to the local contribution of VEGF by the host stroma, which is sufficient to maintain persistent vessels and to sustain tumor growth. When host-derived VEGF is blocked, tumors exhibit extensive necrosis [121]. Breast cancer studies have revealed that mammary stromal fibroblasts might produce factors that influence the growth and malignant progression of a tumor via paracrine effects on the tumor-associated endothelium [122].

### 4.2. The Tumor Recruits Different Types of Cells

Tumor hypoxia caused by the loss of functional vasculature after conventional therapy (e.g., irradiation) results in the upregulation of VEGF to stimulate vascular proliferation and is the stimulus for the influx of BMDCs (bone-marrow-derived endothelial cells). The two principal ways in which a tumor can expand its vasculature as it grows is either by angiogenesis, which involves the sprouting of endothelial cells from nearby normal vessels, or by vasculogenesis, which occurs by the recruitment of circulating endothelial and other cells into the tumor. Both the pharmacological or genetic inhibition of HIF-1*α* attenuates BMDC recruitment and inhibits tumor recurrence. Such BMDC accumulation is composed largely of CD11b+ monocytes. These cells are highly proangiogenic, suggesting that they are attractive targets for enhancing the response of tumors to irradiation [123, 124]. In addition, CD11b+Gr-1+ cells (also defined as myeloid-derived suppressor cells, MDSC) have been found to be frequently increased in tumors and to mediate their resistance to anti-VEGF treatments by producing several angiogenic factors including G-CSF and Bv8 [125]. 

Other cells are important for tumor growth and angiogenesis. For instance, there is an inverse relationship between macrophage density and vascular density [126]. Hypoxia upregulates the production of proangiogenic growth factors and cytokines by tumor-associated macrophages (TAM) [127]. Macrophage infiltration was demonstrated to be a prerequisite step for the angiogenic switch, which correlates with the transition to a malignant tumor phenotype [128]. TAMs secrete a number of mitogenic cytokines and growth factors, which are involved in a range of paracrine loops that promote tumor cell proliferation and growth. A number of studies have shown that TAM infiltration correlates with increased cell proliferation growth of many tumors [129]. The indirect role of TAMs in angiogenesis is also essential for tumor growth, as they provide oxygen and nutrients.

Microglia and macrophages can be recruited either by resident brain microglia or by activated perivascular macrophages. Microglia are recruited to the glioma, where they can produce cytokines to benefit glioma cell proliferation and migration. The cytokines produced include MCP-1 (monocyte chemoattractant protein-1) [130], G-CSF (Granulocyte colony stimulating factor) [131], and several growth factors such as EGF, VEGF, HGF, and SCF [132, 133]. HGF, and its receptor are expressed in both microglia and glioma and stimulates angiogenesis, metastasis and proliferation [134, 135]. Upregulation of TGF*β* might be involved in promoting tumor proliferation and invasion, whereas TNF*α* is mainly produced by microglia because it has been found to be overexpressed in human glioma but not in isolated glioma cell lines [136].

Initially, infiltration of microglia has been proposed to defend the brain parenchyma against tumor cells [137]. However, microglia can interact with the tumor environment and, when activated by the glioma, secrete factors including MMPs that degrade the ECM. Thus, utilizing this strategy, glioma cells can invade and expand into the brain parenchyma [138].

Some characteristics of macrophages/microglia are also exhibited by tumor cells. The phagocytic activities observed in human GBM are properties of malignant macropha ges/microglia [139]. Subpopulations of neoplastic GBM cells exhibit the phagocytic behavior of macrophages/microglia. Notably, GBM tumors contain cells that are positive for both the phagocytic macrophage/microglia marker CD68 and tumor markers such as hTERT. As microglia are the resident macrophages of the brain, subpopulations of the malignant GBM cells could also arise from microglia/macrophages [140].

Myeloid cells have been observed to fuse with tumor cells, producing daughter cells endowed with the invasive properties of myeloid cells and the unlimited proliferative potential of tumor cells (reviewed in [141]). More recently, Pawelek and Chakraborty. demonstrated that the fusion between nonmetastatic cells and macrophages can result in cells with the ability to invade and metastasize [142]. Macrophage/microglial antigens are expressed on neoplastic cells within GBM [143]. It is possible that macrophages fuse with tumor cells during attempts to engulf the cells but that the resulting fusion produces more aggressive and invasive tumor cells [144].

VEGF blockade might be more effective if combined with therapies that also damage endothelial cells. Because endothelial cells proliferate at a slower rate than tumor cells, after the administration of a low-dose cytotoxic therapy, normal endothelial cells might be able to survive during the recovery period [145]. 

Anti-VEGF treatment suppresses the vasculature but not the coopted vessels [146]. Electron microscopic analysis of capillary formation has found that the complex vascular structures within tumors are composed essentially of progenitor endothelial cells. Cells with ultrastructural features of endothelial progenitors are recruited to the tumor periphery prior to vessel formation. Endothelial progenitors are migratory endothelial cells with characteristic ultrastructural features and the capacity to circulate, proliferate and differentiate into mature endothelial cells [147].

Vascular endothelial cells might also represent a target for cytotoxic therapy, as they might be capable of resuming growth during the recovery period after the cytotoxic treatment. However, Browder et al. hypothesized that endothelial cell recovery occurring during this treatment-free period might support the regrowth of tumor cells. This could increase the risk of the emergence of drug-resistant tumor cells [145]. Considering that chemotherapeutic agents themselves can elicit antiangiogenic effects, dosing schedules must be carefully designed to induce maximal apoptosis of the endothelial cells. Some chemotherapeutic agents, in particular, exhibit maximal benefit when administered at a low-dose for long treatment periods (metronomic therapy). The same group has conducted a clinical trial in which children with recurrent or progressive cancers were treated with low-dose chemotherapy in combination with antiangiogenic therapy. Forty percent of patients exhibited prolonged or persistent disease-free status for all of the six months of therapy [148].

Recent studies suggest that tumor cells can also be involved in tumor angiogenesis, as neoplastic lesions have been found to contain tumor-derived endothelial cells (TDECs). These cells originate from the tumor-initiating cells but not from EC progenitor cells. Through the activation of HIF-1*α*, hypoxia plays an important role in endothelial differentiation. This switch is independent of VEGF or FGF. In this model, the VEGF inhibitor treatment elicited no effects on tumor growth [149]. 

Tumors can adapt to treatment with angiogenesis inhibitors by activating alternative angiogenesis-promoting mechanisms to sustain tumor growth [150]. 

In clinical trials evaluating bevacizumab, sorafenib and sunitinib, a minority of individuals failed to show even transitory clinical benefit [151]. In these cases, the tumors exhibited preexisting resistance, which was attributed to the activation of one or more of the aforementioned evasive resistance mechanisms, not in response to therapy but to the selective pressure of their microenvironment. Thus, it is important to identify markers of resistance and to identify new approaches for targeting angiogenesis.

## 5. The Significance and Therapeutic Relevance of ILK1 in the Resistance to Antiangiogenic Therapy

We used Platelet Factor 4-DLR (PF4- DLR), a peptide derived by inserting DLR mutations into a PF4 47–70 aa fragment from Platelet Factor 4 that exhibits strong antiangiogenesis effects and that reduces angiogenesis and tumor growth in a dose-dependent manner in the U87-MG model [152]. This inhibitor has been widely used in human glioblastoma models, in which it significantly inhibits tumor angiogenesis and growth. However, prolonged treatment with PF4-DLR alone or in combination leads to the development of drug resistance, depending on the dose and the tumors stage at which it is administered [153].

Using a proteomics approach, we identified proteins that were differentially expressed in tumors treated with PF4-DLR at two time points: after 10 days of treatment when the tumors are responsive to the antiangiogenic therapy, and after 20 days of treatment when glioblastomas are still responsive to PF4-DLR. However, if treatment is prolonged, glioblastomas start to activate new pathways that might induce drug resistance. The significance of Integrin-linked kinase 1 (ILK1) expression after PF4-DLR treatment was investigated in greater detail. Interestingly, we found that ILK1 expression is downregulated after 10 days of treatment and upregulated after twenty days. This result suggested that ILK1 expression correlates with treatment response, at least in our experimental model. ILK1 is a protein that is involved in intracellular signal transduction of integrins and growth factor receptors. In some tumors, increased ILK1 levels are required for cell growth/survival, cell cycle progression, invasion and migration, and tumor angiogenesis [154]. In glioblastoma, a link between ILK1 and tumor cell invasion has been proposed [155]. Over time, ILK1 increases the expression of VEGF, implying that ILK1 might be a key molecule for a positive signaling loop that induces angiogenesis and tumor growth. Edwards et al. demonstrated that inhibiting ILK1 with small molecule inhibitors reduces tumor hypoxia, decreases tumor vascular mass and decreases functional vasculature in a mouse model of glioblastoma [156]. The inhibition of ILK1 alone was able to delay but could not completely inhibit tumor growth. We therefore decided to inhibit ILK1 using siRNA in addition to PF4-DLR administration in order to investigate whether this combinatorial approach would further improve therapeutic efficacy *in vivo* over PF4-DLR treatment alone. Interestingly, treatment with PF4-DLR and an anti-ILK1 siRNA resulted in decreased tumor mass and a reduction in the number of tumor vessels. Our findings have important therapeutic implications and suggest that combinatorial strategies that simultaneously inhibit different mechanisms of tumor proliferation and angiogenesis might significantly increase therapeutic efficacy. We also analyzed the ILK1 expression levels in patients with glioblastomas, astrocytomas and oligodendrogliomas and found that high levels of ILK1 expression correlate with poor prognosis. Our data suggest that ILK1 could represent a novel specific pharmacological target to be inhibited alone or in combination with antiangiogenic therapies for gliomas [157].

## 6. Future Perspectives

The VEGF family members, angiopoietins, Notch/Delta4 or platelet-derived growth factor are currently the major focus of angiogenesis research. However, many other regulatory molecules, including chemokines, critically modulate vessel growth. The inhibition of IL-6 and VEGF results in the inhibition of U-87-derived experimental glioma growth on chick CAM (chorioallantoic membrane) or in xenografts in the brains of mice [158].

If a tumor depends on the activity of a single kinase, a new approach is to target the overactive kinase using multiple drugs. However, prolonged therapy can again select for mutations that give rise to therapeutic resistance of the tumor [159, 160]. 

An alternative strategy is to target groups of different kinases. This approach has been demonstrated to be effective in animal models and is undergoing clinical testing. At least 21 clinical trials are currently evaluating the combination of a tyrosine kinase inhibitor and an mTOR inhibitor in several different types of cancer. 

The specific targeting and delivery to malignant cell populations can be achieved by targeting cell surface receptors that are either uniquely expressed or overexpressed on cancer cells. A variety of ligands have been evaluated for this purpose, alone or affixed to nanoparticles, antibodies and related fragments, other proteins, peptides, or aptamers. On the basis of the discovery that most human tumors express a high density of specific receptors, it has been possible to develop radiolabeled peptides that localize to these tumors and their metastases for therapeutic targeting [161]. For example, NP-Apt (nanoparticle-aptamer) bioconjugates have demonstrated therapeutic efficacy both *in vitro *and *in vivo *against cancer cells. 

Modular nanotransporters (MNTS) are recombinant multifunctional polypeptides created to exploit a cascade of cellular processes, from the initiation of membrane receptor recognition to the delivery of selective short-range and highly cytotoxic therapeutic agents into the cells. Slastnikova et al. demonstrated that MNT can selectively deliver drugs into the nuclei of tumor cells that express a targeted receptor, thereby improving the specificity of the therapeutic drugs [162].

Aptamers are oligonucleic acid or peptide molecules that can bind to target molecules (nucleic acids, proteins, small molecules, and even cells) expressed on both on membrane surfaces and intracellularly. Aptamers are more advantageous than antibodies because are easy to synthesize and can be conjugated to fluorescent dyes or chemically modified to exhibit low immunogenicity. However, a major limitation in their clinical application is their low bioavailability after administration.

Aptamers have been used both as a tool to find new targets in cancer therapy and as therapeutic agents against tumor angiogenesis [163]. In December 2004, the US FDA approved pegaptanib (Macugen), an anti-VEGF RNA aptamer, for the treatment of all types of neovascular age-related macular degeneration. Pegaptanib is the first aptamer to be successfully developed as a therapeutic agent for clinical application in humans[164].

## Figures and Tables

**Table 1:** A critical summary of the major *in vitro* and *in vivo* models to study tumor angiogenesis.

Cellular models	Characteristics	References
Human tumor cell lines	There are a total of 60 cell lines representing nine distinct tumor types. However, this model does not reflect the complexity of the real tumor environment.	[6, 7]

Multicellular tumor spheroids	Genes associated with cell survival, proliferation, differentiation, and resistance to therapy are differentially expressed in cells grown as multicellular spheroids versus 2D cultures.	[8–13]
	The capacity for spheroid outgrowth in 3D matrices is an interesting parameter to study the migratory behavior of tumor spheroid cells; however, this parameter can only be used for rapidly migrating cells (e.g., glioblastoma spheroids).	
	Endothelial cell spheroids are increasingly used for evaluating the pro- and anti-angiogenic potential of drugs.	
	Cospheroids of HUVEC and human fibroblasts are used for angiogenesis studies.	
	Tumoral spheroids cocultured with endothelial cells potentiate tumor angiogenesis by upregulating proangiogenic factors that are absent in multicellular tumor spheroids alone or in monolayers.	
	Another advantage is the possibility to use tumor spheroids from biopsies. This is useful for the study and development of patient-specific therapies and for the presence of tumor-initiating cells and tumor progenitors stem cells in tumor spheroids.	

Xenograft models	Characteristics	References

Chicken chorioallantoic membrane tumor assay	The CAM tumor model could allow for a prescreening of drugs and subsequently reduce the number of animals used for *in vivo* experiments. This model is much faster than animal models. Histological analyses of the CAM tumors revealed a well-organized tumor tissue that strongly resembled clinical specimens of human tumors. The CAM model allows for the formation of tumors comparable to patient samples, with a degree of fidelity to human disease that is impossible to achieve with other nonanimal models; it combines the advantages of an *in vivo* environment with the simplicity of an *in vitro* experiment
	The duration of the follow-up period is limited due to the hatching of the chick 21 days after incubation.	[14]

RG2 and F98 rat cell lines	Tumors were produced by Wechsler in Koestner'slaboratory by the i.v. administration of a single dose of ethyl-nitrosourea (50 mg/kg b.w.) to a pregnant CD Fischer rat on the 20th day of gestation. The isolated clones retain individual characteristics, including the differentiation status, despite repeated propagations *in vitro*, elevated mitotic index and an increased nuclear-cytoplasmic ratio consistent with glioma cells in culture. When injected, these tumors have been refractory to chemotherapy and radiotherapy and adaptive to immunotherapy and exhibit an infiltrative pattern of growth within the brain. These characteristics closely resemble those of human glioblastoma.	[15]

Subcutaneously implanted human tumor xenografts	Tumors obtained from the direct implantation of the human cell lines or patient tumor biopsies are models that allow the monitoring of tumor growth. However, growth can be too slow; in xenografted models, the microenvironment and host immune responses are altered, and this may influence the tumor response.	[16, 17]

Orthotopic xenograft models	This model mimics the morphology, growth characteristics of clinical disease and metastatic processes more efficiently. There are several studies that report differences in the therapeutic responses between subcutaneous and orthotopic models.	[18, 19]

J3T-1 and J3T-2 orthotopic mice and rat models	The traditional orthotopic models for brain tumors did not aggressively invade healthy brain tissues; for this reason, we do not have an ideal GBM animal model that incorporates all of the human GBM features. Spontaneous canine glioblastoma approximates the human disease characteristics. However, it is not trivial to study a large number of spontaneous canine glioblastomas. The orthotopic xenograft implant of the two GBM cell lines, J3T-1 and J3T-2, into immunosuppressed mice and rats histologically recapitulated two invasive and angiogenic phenotypes: angiogenesis-dependent and angiogenesis-independent invasion observed in human glioblastoma.	[20, 21]

Spontaneous/genetic models	Characteristics	References

Pten-, Rb1-, Tp53-deleted mice	The HGA murine models with *Pten, Rb1, *or *Tp53 *deletion are relevant to human disease, reflecting a spectrum of tumor histology and molecular features. Thus, molecular and other complex processes including specific contributions of the tissue microenvironment, such as tumor angiogenesis, can appropriately mimic human disease in these spontaneous tumor models.	[22, 23]

VM-M3 spontaneous tumors of the VM mouse strain	The inbred VM mouse strain is unique in exhibiting a relatively high incidence (1.5%) of spontaneous brain tumors. The VM-M3 brain tumor arose spontaneously in the forebrain of a VM mouse and expresses properties of microglia/macrophages similar to that seen in several types of invasive cancers of neural origin. Similar to high-grade human gliomas, the VM-M3 tumor cells, highly invasive, can be grown in the syngeneic VM mice with reproducible growth rates and have genetic similarities to human GBM. In addition, the tumor cells are labeled with the firefly luciferase gene allowing for noninvasive detection and quantitation of tumor growth.	[24]

Canine spontaneous glioma	GBM is the most common primary brain tumor in dogs and brachycephalic breeds such as Boston terriers and Canine Boxers are genetically predisposed to develop these tumors. Spontaneous gliomas may provide a valuable large animal model for the investigation of novel delivery and therapeutic strategies for intracranial tumors. The presence of pseudopalisading necrosis and endothelial proliferation that closely resemble those found in human GBMs suggests the presence of a hypoxic environment in canine GBM. The large size of the canine brain compared to the rodent brain would be more useful for preclinical assessment of doses, comprising more relevant volumes needed to implement novel therapies. However, spontaneous GBM in dogs is not a tumor model that is as easily accessible as the rodent GBMs. These models have variable penetrance, resulting in lack of synchrony in tumor development. The variability in time to progression represents a limitation in its use for drug testing.	[20, 25, 26]

F98 rat glioma	This tumor is produced by the administration of a single i.v. dose of ethylnitrosourea to a pregnant rat. It has been classified as an anaplastic and undifferentiated glioma. It is refractory to chemotherapy and radiotherapy. This model is effective for the evaluation of survival rate. However there are serious limitations in directly applying data from rat tumor models to any clinical treatment for human brain tumor.	[15, 27]

**Table 2:** Summary of the available treatments and the relative clinical phase and results.

Drug	Target	Clinical phase	Results
Endostatin (Endostar)	Interfere with the proangiogenic action of growth factors	Phase III 2005	Significant and clinical improvement in response rate, median time to tumor progression, and clinical benefit rate in combination with chemotherapy [67].

Bevacizumab (Avastin)	Monoclonal antibody anti-VEGF	Approved in 2004	In May 2009, the FDA approved Avastin as a single agent for the treatment of recurrent GBM based on the demonstration of objective response rates in two single-arm trials: AVF3708g and NCI 06-C-0064E.

Cilengitide	Selective inhibitor of *α*v integrins	Orphan drug by European medicines agency in 2008	Phase II trial in conjunction with chemotherapy and radiation: EMD 121974 in 2010 phase II trial in recurrent glioblastomas. The efficacy of the cilengitide alone is modest, but it is adequately delivered to the tumor [68]. In a phase II study, the addition of cilengitide to standard chemoradiotherapy demonstrated promising activity in GBM [69].

Etaracizumab (Abegrin)	Humanized monoclonal antibody direct against the human *α*v*β*3 integrin	Phase II/phase I	Well tolerated with no evidence of immunogenicity [70]. Does not improve the effect of dacarbazine in a phase II trial of metastatic melanoma [71].

Volociximab	Chimeric monoclonal antibody that binds to and inhibits *α*v*β*1 integrin	Phase II	Despite insufficient clinical activity in the refractory patient population to continue the study, weekly volociximab was well tolerated. A better understanding of the mechanism of action of volociximab will inform future development efforts [72].

Marimastat	Broad-spectrum matrix metalloproteinase inhibitor	Phase III	Treatment with marimastat in SCLC and GBM patients does not improve survival [73, 74].

Sorafenib	Small molecular inhibitor of several tyrosine protein kinases (VEGFR and PDGFR) and Raf kinases	Approved in 2007 for liver and kidney cancer	Phase I and II trials for brain tumors. Sorafenib can be safely administered [75, 76].

Cediranib	Potent inhibitor of VEGFR	Phase I, Phase II	Modest single-agent activity [77, 78]. Cediranib monotherapy yielded encouraging responses in recurrent glioblastoma in a phase II study [79].

Sunitinib	Multi-target receptor tyrosine kinase inhibitor	Approved for renal cell carcinoma and for imatinib-resistant gastrointestinal stromal tumor	Single-agent sunitinib exhibited insufficient activity in patients with recurrent glioblastoma in a phase II study [80].

Imatinib	Specific inhibitor of receptor tyrosine kinase	Approved in 2011 for ten different cancer types	In brain tumors, it did not show clinically meaningful antitumor activity in phase II and phase III trials [81–83].
